# The Principles and Practice of Obstetrics

**Published:** 1858-05

**Authors:** 


ART. III.—*The Principles and Practice of Obstetrics; Including the Treatment of Chronic Inflammation of the Uterus, considered as a Frequent Cause of Abortion.* By HENRY MILLER, M.D., Professor of Obstetric Medicine in the Medical Department of the University of Louisville. With illustrations on wood. 8vo., pp. 620. Philadelphia, 1858. Blanchard & Lea.

NEARLY nine years ago, Dr. Miller published, at Louisville, a treatise on human parturition, which may be considered, to some extent, as the nucleus of the present work. That treatise, though fragmentary and confessedly incomplete, exhibited, nevertheless, not merely an intimacy with the several subjects discussed in it, but, in respect to most of them, an originality of thought, and throughout, a disregard of mere authority in reference to such questions as are open to investigation alike to every one engaged in the active duties of obstetrical practice. It was, in consequence, most favorably received, and a desire expressed that Dr. Miller might be induced to carry out and perfect the plan, somewhat roughly sketched in the publication referred to, by the preparation of a regular and complete treatise on the theory and practice of midwifery. This task he has at length accomplished, by the publication of the work before us; the execution of which confirms the most favorable of the anticipations based upon the character of the former treatise. We find in it the same disposition manifested on the part of the author, to employ his own faculties and opportunities for observation in the investigation of every question of doctrine, and his own judgment, enlightened and confirmed by the results of his personal experience, compared with those of the recorded experience of others, in determining correct rules of practice. The same fearless announcement of his own conclusions, when convinced of their accuracy, however much they may be at variance with received opinions and practical dogmas, or opposed to the cherished views of some would-be obstetrical autocrat. In the reasons—always plausible and often conclusive—adduced by Dr. Miller in evidence of the correctness of his dissent, on any given question, from the teachings of distinguished obstetricians, he evinces far more ardor in the cause of truth than desire to establish a fictitious title to originality in either doctrine or practice.

The foregoing remarks are made in perfectly good faith. We believe them to be strictly applicable to the work before us. At the same time, we should not wish to be considered as unduly exalting its merits, or as affecting to see in it neither defect, fault, nor omission. The treatise of Dr. Miller is certainly far from being complete or perfect. It fails, in the first place, to cover the entire ground demanded by its title: more than one subject intimately connected with the theory and practice of obstetrics being entirely omitted; while others, though treated of, have not that prominence and fulness of exposition awarded to them which their importance demands. We object, also, to the arrangement of the work, as one not well adapted for the instruction of the student, nor for easy reference, as occasion may demand, on the part of the practitioner. It is true, the arrangement of subjects being, in a good degree, that in which they usually occur in practice, or, at least, in accordance with their most ordinary clinical relationship, it may, in some respects have its advantages, especially in the case of the advanced student and young practitioner, who will be willing to sit down to the study of the treatise as a whole, rather than to the study of either of the questions of theory or practice, embraced in the general subject, individually, or detached from their connections. The style of Dr. Miller, though it has but slight claims to elegance or purity, is, nevertheless, sufficiently clear and vigorous; on more than one occasion, however, it is disfigured by modes of expression which ought to be carefully excluded from a grave didactic treatise on a subject strictly scientific; while, occasionally, the tone indulged in by the author, when controverting the opinions of others on particular questions, is marked by a degree of apparent harshness, calculated to mislead the reader into a belief that the opinions objected to have been attacked as much on account of those who uphold them as from any real or supposed error in the opinions themselves. Still, with all these defects and blemishes—and the list might probably be enlarged—we pronounce the work of Dr. Miller an able and valuable one,—one destined to assume a high and permanent rank among the standard American treatises on the theory and practice of midwifery. It is not only generally full, correct, clear, and distinct in its teachings, but it presents a manly vigor and directness in the handling of all the questions embraced within its general subject, that cannot fail to impart themselves, in some extent, at least, to its readers; inducing them, in their turn, to examine, think, and decide for themselves—to seek for truth where alone truth can be found—in the field of cautious observation and carefully studied experience; not, however, forgetting the guidance, whether suggestive or by way of comparison, of such facts as have been carefully recorded by reliable observers.

It is not necessary that we should enter into an examination of the first portion of the treatise, or that devoted to a description of the physical structure of the pelvis, its regions, measurements, planes, and axes; the anatomical structure and normal functions of the female sexual organs. Suffice it to say, that these subjects are treated with that fulness and accuracy which their important bearing upon the doctrines and practice of obstetrics demand. Without an intimate acquaintance with every particular included in these preliminary chapters, it is impossible to understand the first principles of obstetrical science, or to become a successful practitioner. Without such acquaintance it is in vain to attempt the safe conduct of even the simplest case of natural labor.

For the anatomical description of the female pelvis and organs of generation, and their obstetrical relations, the author acknowledges his indebtedness to the recent work of M. Dubois, of Paris. It was scarcely possible to obtain from any other source a more full, clear, correct, and, at the same time, concise account of these subjects. It is precision and accuracy that are to be aimed at in mere anatomical descriptions: in our account of physical structure—form, regions, diameters, planes, and axes, and the relation which these bear to each other—originality, even if it were attainable, is of secondary importance.

The preliminary matter referred to occupies the first two chapters of the treatise. It is well and copiously illustrated by excellent drawings and diagrams.

The third chapter is devoted to an account of the clinical exploration of the female sexual organs, not simply in reference to the question as to the existence or non-existence of pregnancy,—which is but slightly touched upon,—but for the purpose of detecting the presence of any deviation in the position of the uterus, or any abnormal condition of its tissues or annexes. The entire subject is very ably treated; but we very much doubt the advantage of this extension, in a treatise like the one before us, of the field of uterine exploration beyond its strict obstetrical limits. The title-page of the present treatise announces, it is true, that, besides its leading subject—the principles and practice of midwifery—it includes, also, "the treatment of chronic inflammation of the uterus, considered as a frequent cause of abortion." We admit, likewise, that, to the obstetrician, a knowledge of the pathological conditions of the womb and its appendages is all-important, inasmuch as these conditions are liable to interfere with the regular course and favorable termination of utero-gestation; still, we maintain that an acquaintance with the etiology, diagnosis, and treatment of uterine diseases is to be acquired from the proper source;—that the consideration of their pathology, symptomatology, and therapeutical management is out of place in a treatise on the science and practice of obstetrics. With equal propriety might an account of several of the acute diseases of the general system, and other affections in which the uterus is occasionally and indirectly implicated, be introduced into our systematic treatises on obstetrics, upon the plea that they are among the frequent causes of abortion and premature labor. In the discussion of the interesting question as to how far chronic inflammation of the uterus is to be considered a cause of abortion, it was not at all necessary that Dr. Miller should interrupt the regular course of his treatise for the purpose of entering as fully as he has done into the general subject of uterine pathology and therapeutics.

The subject of the ensuing, fourth, chapter is pregnancy. The prelections of Dr. Miller on this all-important branch of the physiology of the female are marked by great ability. The phenomena of pregnancy, both maternal and fœtal, from the period of conception to the completion of utero-gestation, are clearly and accurately delineated; while the important questions that have arisen in respect to the nature of the successive changes that take place in the mucous membrane, vascular system, muscular tissue, and in the vital condition, size, figure, and relations of the womb during the period of gestation, as well as in reference to the true character of the intra-uterine life and nutrition of the fœtus, and the true connection existing between its organism and that of the mother, are carefully examined and elucidated by the results of the most recent and reliable facts and observations.

Dr. Miller examines the validity of the position assumed by Levret, Baudelocque, and others, and, what is most remarkable, still advocated by distinguished obstetricians of our own time: namely, that the development of the uterine cavity, in the course of pregnancy, is the result of the growth of the ovum. The fundus giving way before the distending force of the latter first, subsequently the body, and finally, the cervix; so that, toward the close of gestation, the fundus, body, and neck of the womb, form but one common cavity, nothing remaining of the cervix but the cushiony circle of the external or vaginal orifice. In connection with this doctrine of a forced development of the uterus, it was also held, and still is by teachers of high authority, that during pregnancy an antagonism exists between the muscles of the body and those of the neck of the uterus; in consequence of which the former tend constantly to expel the ovum from the uterine cavity, while the latter, with an equal force, resist its expulsion. From this equilibrium between the two forces, in the normal condition of things during gestation, the ovum is retained, until toward the close of pregnancy, when the muscles of the body of the womb which were first developed to their full capacity, offer the greatest resistance to any further distension; the equilibrium between them and the muscles of the neck is, in consequence, destroyed. The fibres of the body begin now to make efforts to expel the fœtus; and, henceforth, the fibres of the neck, receiving the entire distending force of the uterine contents, as well as the reaction of the body, are rapidly developed, until, finally, labor sets in, and the expulsion of the fœtus is no longer resisted by the extenuated muscles of the cervix.

In respect to these very clumsy and purely mechanical views, Dr. Miller remarks:—

"It is manifest that the theory rests on the assumption that the uterus is naturally disposed to resist, *unguibus et rostro*, all intrusions upon its premises, an assumption so contrary to analogy that it ought not to be admitted without the clearest proof. But let us examine its pretensions in its twofold application; and *first*, as affording an explanation of uterine development. Here it seeks to expound a phenomenon which is itself gratuitously assumed, namely, such a development of the cervix as enlarges the capacity of the cavity of the gravid uterus.

"According to the observations of M. Cazeaux, the neck, especially in women who have borne children, preserves the whole of its length until the last fifteen days of pregnancy, or at least until the commencement of the ninth month. He avers that he has repeatedly verified this fact, which had been already noted by Professor Stoltz, of Strasburg, and publicly taught by Professor Dubois since 1839.

"In primiparæ, the cervix uteri offers some peculiarities, which, as far as our present subject is concerned, consists in its shortening somewhat, instead of preserving its usual length, throughout the greater part of pregnancy, as in multiparæ, and in its internal orifice becoming dilated before the external. Professor Stoltz explains this shortening in the following manner:—At the sixth month, the vaginal portion of the neck begins to shorten, *while it widens at its superior part.* The external orifice, continuing closed, approaches the internal, and consequently the cavity of the neck becomes larger in the middle, *until the two orifices are brought near each other; the internal orifice then opens first,* which happens during the last fifteen days of pregnancy; the rest of the body disappears much more rapidly than it had done before, and a projection can no longer be felt: the external orifice remains closed.

"The observations of Professor Stoltz are substantially confirmed by M. Chailly, and the entire account which he gives of the changes that the neck undergoes during pregnancy, contradicts the hitherto received opinions of writers on the subject.

"There is one well-known fact to which we may allude, that goes far to establish the accuracy of these researches of M.M. Stoltz, Dubois, Cazeaux, and Chailly, if it be not, of itself, sufficient to refute the opinion formerly entertained. It is this:—When the neck of the uterus is so much developed as to allow the finger to be passed to its upper orifice, which it is by the seventh month in multiparæ, the membranes can be felt, and are organically united to the uterus around the margin of the orifice. When, again, the neck is entirely obliterated, as it is at term, the membranes can be felt, and are still attached around the os uteri. Now, as it is admitted that, during the first five or six months, the ovum is confined to the cavity of the body, and that the neck is not lined with decidua, were the obliteration of the neck owing to the expansion of its upper part, either the membranes would be too high to be reached by the finger, or if they were sufficiently extensible to be pushed down into the expanding neck by the growing ovum, they would not be found adhering to its surface. The latter declaration is authorized by the fact that, in the progress of gestation, the membranes become less vascular, and their adhesion to the internal surface of the uterus is gradually weakened. But, in either case, at the seventh or ninth month the membranes are found to have vascular connection around the uterine orifice, for when separated by the finger, or by uterine contractions, as in the latter case they are so soon as labor commences, there is a slight effusion of blood. We conclude, therefore, that the neck contributes nothing to the cavity of the gravid uterus, which is made up entirely of the dilated cavity of the body.

"The *secondary application* of the theory to explain the induction of labor is best met by demanding the proof that the uterus displays, during gestation, such intolerance of its burden as is attributed to it.

"There is absolutely no evidence of such contractile efforts of the uterus as this theory assumes, except the occasional tension of the membranes, sometimes observed toward the completion of gestation, the os uteri being then sufficiently open to admit the finger. Slight contractions of the fundus may produce this tension, but these are not such as constitute labor, for they are unaccompanied by pain, and take place without the consciousness of the individual herself. Allowing, however, that they are labor-pains in disguise, their presence at so advanced a period of pregnancy is no proof of their existence during the earlier periods; and in complete absence of such proof, we are loath to admit the assumption that they do exist, because it makes the uterus the strangest anomaly in the body, if not in nature. It is destined first to contain and nourish the fœtus, and then to expel it when its maturity is acquired. But, according to this assumption, the first is an irksome task imposed upon it, which it continually endeavors to quit by expelling its contents. Such a constitution of the gestative organ could hardly exist, and abortion be not perpetually threatened, without, as far as we can perceive, any compensating benefit; for we cannot imagine that its development could be promoted by it. There is, in fact, no conceivable way in which contraction of the uterine fibres during pregnancy could favor their development, except that imagined by Baudelocque, viz., one class of fibres stretching another by the superior force of their contractions, by which he attempts to account for the development of the cervix uteri. How then are the fibres of the body of the uterus developed during the first six or seven months of gestation, the neck being quiescent all the while? If those need no such force to aid their development, neither do those of the neck; both are developed after their own peculiar fashion, without the interference of one with the other. The neck, as we have seen, is developed in women who have borne children, in a manner inconsistent with the idea that any sort of force is exerted upon it by the body; that is, from below upward.

"From what has now been declared, it may be inferred that I reject the notion of Levret, endorsed by Baudelocque, and more recently by Meigs, that the neck is the antagonist of the body of the uterus, *during pregnancy*, simply for the reason that there is not a tittle of evidence that there is anything to be antagonized. The body quietly suffers itself to be distended by the product of conception, or rather its growth keeps pace with that of the ovum, while the neck is not at all concerned either in making room for the fœtus or barring its escape. But though the neck be not called on to exert a retentive faculty, it holds, nevertheless, the key, if I may so say, which unlocks the uterine cavity, by virtue of the relation established between it and the body by pregnancy, in consequence of which impressions made upon its internal surface are reflected upon the body and excite its muscular fibres to expulsive action. Hence the necessity of keeping the neck closed to so late a period of pregnancy, while the body is growing and expanding in every direction. That labor is naturally excited in this way I shall endeavor to prove hereafter. The neck is not the active but the passive custodian of pregnancy; it simply withholds the key, until the time to unlock the uterus has arrived."

That the enlargement of the uterus, during the progress of gestation, is an active process, an actual growth, and not a mere passive distension of its parieties, the result of the pressure exercised upon them by the contained ovum, may be considered as an established fact. That under all the normal conditions of pregnancy, no disposition is evinced on the part of the uterus to expel its contents, and, consequently, no necessity for any power on the part of the muscles of the uterine neck to resist this expulsion until the period of gestation is accomplished, must, we think, be evident to every one who will take the trouble to examine the subject with care. The office ascribed by Dr. Miller to the cervix uteri is, it is true, a hypothetical one,—it has nevertheless all the characteristics of truth, and is in close accordance with established facts and observations.

The history of the ovum, from its first introduction into the uterine cavity until the completion of its placental connection with the maternal organism, as given by Dr. Miller, is particularly interesting. In relation to nearly everything connected with the early life of the ovum in utero, the views heretofore almost universally entertained, have been shown, by recent and more carefully conducted observations, to be incorrect.

In place of the ovum acquiring what has been hypothetically termed its reflex decidua, by encountering at the uterine orifice of the Fallopian tube the decidua vera, which is stretched across the orifice, and is pushed before it by the ovum as it enters the cavity of the uterus, forming at first only a slight protrusion in the cavity of the true decidua, which increases, however, with the growth of the ovum, until eventually it comes in contact at all points with the uterine walls, which are then lined by two layers of decidua. Instead of this, which is the Hunterian hypothesis, it will be found, upon examination, that the minute fecundated ovum after its entrance into the cavity of the womb becomes imbedded in the lining membrane of the latter, which has become prepared for its reception by an increase of vascularity, thickening, and softening, and which quickly grows up around it, and encloses it between two layers of membrane, the outermost being the so-called *decidua vera* and the innermost the *decidua reflexa* of Dr. Wm. Hunter; and which Dr. Miller suggests to designate as *uterine* and *fœtal* decidua.

During the first two months of pregnancy the ovum is attached to the decidua by the villi of the chorion, forming a number of slender cords, "which," as Jacquemier finely expresses it, "is the most delicate mode of suspension, and best calculated to prevent injurious consequences from concussions, blows, &c., at a period when protection is most needed. They are, also, spongioles for absorbing nutriment from the surrounding fluids of the mother, besides being essential elements in the construction of the placenta."

It is not until the third month of pregnancy that the placenta begins to make its appearance upon that portion of the chorion which is toward and in contact with the uterine decidua. The place at which the ovum becomes connected with the uterine membrane, first by the villi of its chorion, and subsequently through the intermedium of the placental structure, is variable. The ovum may become imbedded in the mucous coat of the uterus soon after its entrance into the womb, and consequently in the neighborhood of one of the Fallopian orifices, or it may be carried some distance from thence before its arrest and fixation.

In reference to the nature of the placental connection between mother and fœtus, the views advanced by Weber are adopted by Dr. Miller, namely, that a congeries of colossal capillaries intervene, into which the utero-placental arteries open, and from which the utero-placental veins arise. Against these capillaries, in which the circulation is extremely sluggish, the villi of the chorion impinge, and, as it were, penetrate, as they prolong their growth outwardly, and become, as must necessarily be the case in such a process, invested with a covering of the inverted membrane, forming the walls of the decidual capillaries.

In regard to the much-disputed question, What office does the placenta fulfill in respect to the fœtus? Dr. Miller replies, and we believe correctly, that the office of the placenta is twofold, being first, the organ of respiration of the fœtus, and, in the second place, the organ through which the fœtus derives its nourishment from the mother.

> "The umbilical vessels," he remarks, "already described as terminating in capillaries upon the dentritic processes of the chorion, belong exclusively to the vascular system of the fœtus. They consist, as we have seen, of three trunks, two arteries, and one large vein—a branch of the inferior vena cava—and have no communication whatever by anastonosis with the blood-vessels of the mother. The arteries convey no inconsiderable portion of the blood of the fœtus to the placenta, which, after circulating freely and minutely through it, is returned to the fœtus, not a drop passing into the vessels of the mother. While circulating in the placenta, this blood is brought in contact with the blood of the mother, flowing through the canals of the maternal portions of the placenta; or, at least, nothing intervenes but the thin walls of these canals, and the delicate coats of the fœtal capillaries. The fœtal blood is thus enabled to abstract oxygen from, and impart its superfluous carbon to, the blood of the mother; and although it may be supposed that this vital operation is not as freely performed as in animals that inhale atmosperic air, it is at least as advantageous an arrangement as the branchial respiration of such as inhabit the water, to which it is, in fact, analogous—fishes getting their oxygen from water, and the fœtus from the maternal blood."

Dr. Miller adduces the consequences which result from compression of the cord to such a degree as to arrest the circulation of the blood through its vessels, as conclusive proof that the fœtal blood is aerated in the placenta. We know that such compression is liable to happen during labor, when the cord prolapses before the head of the child in vertex presentations, and during the passage of the head through the pelvis in presentations of the nates, and that whenever it does, speedy death is the consequence, attended by all the phenomena of suffocation.

In proof that the fœtus derives its nourishment from the mother through the placenta there is, it is true, no positive evidence; Dr. Miller insists, however, that the negative evidence bearing upon this point is altogether satisfactory.

"There is," he remarks, "absolutely no other medium through which the fœtus can obtain its supplies of alimentary matter. The only other possible source is the liquor amnii,—the fluid which surrounds the fœtus; and the doctrine that this is appropriated, either by absorption or deglutition, has long since been exploded by facts and arguments that cannot be answered, which need not be rehearsed in this place. How, or in what form nutriment is received through the placenta is not known. Most probably there is a set of vessels, in connection with the umbilical capillaries, which open into the maternal portion of the placenta, and, abstracting from thence the needful supplies, convey them at once into these capillaries, to be incorporated with the fœtal blood. Whether these hypothetical vessels take up blood, or only certain of its elements, we do not know, nor, as far as I can see, is it a matter of the least practical moment that we should know. Nature, here as elsewhere, is chary of her revelations, that might gratify the curiosity without adding to the resources of her votaries."

A portion of the concluding section of the present chapter, which treats of the position of the fœtus in utero, is somewhat, as we conceive, unprofitably occupied with an inquiry into the cause of the great preponderance of vertex presentations, and a long exposition of facts to prove the incorrectness of the supposition, still admitted by some, and at one period very generally entertained, that it is due to the greater weight of the head over that of the rest of the body, throughout every stage of fœtal development. Dubois and Simpson ascribe the greater frequency of head presentations to a vital power inherent in the fœtus; the usual position of which, according to the first, is assumed and maintained by its own voluntary or instinctive movements; while, according to the latter, it is the result of a purely reflex or excito-motory action. Of this vital doctrine Dr. Miller is an adherent.

"Whether," he remarks, "with M. Dubois, we regard the fœtus as the subject of sensation and voluntary motion, or, with Professor Simpson, consider it as insensate, but capable of as lively excito-motory phenomena as a decapitated frog, we may account, upon either theory, for its taking up its usual position in the womb during the latter months of pregnancy, and recovering it when lost by the operation of any disturbing cause. In any other position, there is a want of adaptedness to its lodging, and the pressure made upon its surface by the opposing uterine parieties, whether it acts as an excitor-stimulus, awakening reflex motion, or produces disagreeable sensations, leading to sensorio-volitional movements, is followed by a series of movements which do not cease until the fœtus has recovered its adaptive position, in which it is freed from such stimulus or disagreeable sensation. It is not so in the early months of gestation, when, as we have seen, there is ample scope for the fœtus in the relatively larger cavity of the womb, and it may assume any position it pleases, (if it can be pleased;) hence, the greater proportionable number of mal-presentations in cases of premature labor, the fœtus being caught in its gambols."

This is all pure hypothesis—a mere guessing at what we know nothing about; and without the remotest advantage in a practical point of view. As Dr. Miller himself admits, the most frequent intra-uterine attitude of the fœtus, with its limbs flexed and folded upon its body, and its head dependent, is "among the most felicitous of its obstetric aptitudes," adapting it in form to that of the uterine cavity in the advanced stage of pregnancy, and presenting it under the most favorable aspect for the proper progress and safe completion of the mechanism of labor. It is a legitimate conclusion, therefore, that it is the attitude impressed upon the fœtus by the same vital laws which govern everything relating to intra-uterine gestation. As to the precise manner in which these laws act in producing and maintaining what may be assumed as the normal position of the fœtus, or what are the forces by which the operation of those laws are occasionally interfered with, and mal-presentations allowed to occur, we are and will probably ever remain in profound ignorance.

The subject next treated of is abortion, (Chap. V.) It is a subject of deep interest, and from the frequency of its occurrence, and the actual danger with which, in the majority of instances, it is attended, demands the closest study on the part of the obstetric practitioner; a large share of whose attention should be directed to the investigation of its most frequent causes, with a view to fix upon the means best adapted to prevent its occurrence; to arrest, if possible, its progress, so as to secure the safety of the fœtus, or, if this be impossible, to protect from injury the constitution of the female in whom it takes place.

One of the most constant attendants upon abortion, and a chief cause of the immediate danger attendant upon its occurrence, is profuse or long-continued hemorrhage. According to Dr. Miller, the source of this hemorrhage is wholly different from that which attends or immediately follows the extrusion of the secundines in natural parturition. The blood in abortion proceeding chiefly from the decidual vessels, probably from both layers of the decidua; it may issue from any point of the ovum or internal surface of the uterus, while in parturition it flows exclusively from that portion of the uterus to which the placenta was attached, and proceeds from the ruptured utero-placental vessels. He believes that in abortion the hemorrhage is often the result of decidual hyperæmia,—an exudation from the distended and overloaded blood-vessels of the decidua, without rupture. This, he thinks, may be inferred from the sense of fulness and weight which is not unfrequently complained of previous to the eruption of blood, as well as from the further fact that the hemorrhage may precede the uterine contractions, and is always coeval with them, even in their incipiency, when they are too feeble and few to disturb the relations of the ovum to the internal surface of the uterus.

These views of Dr. Miller are highly plausible, and demand, at least, a candid examination, in conjunction with the evidence adduced by him in their support.

After a very good sketch of the symptoms and signs of abortion, its causes are considered. These are admitted to be multifarious. It may be the result of various accidental causes, such as injuries and strong mental emotions; of febrile diseases, especially the exanthemata; of drastic purgatives, or direct uterine stimulants; of diseases of the ovum, resulting in the death of the fœtus. But, according to Dr. Miller, the most prolific cause of abortion is a diseased state of the gestative organ itself, especially inflammation, either of the cervix, or of the entire organ. He considers that—

"Inflammation of the mucous membrane of the body of the uterus may, doubtless, directly excite contractions which shall expel the ovum, just as inflammation of the intestinal mucous membrane may directly excite increased peristaltic movements which shall expel the feces; but when the inflammation is limited to the cervix uteri—and this is most frequently the case—it acts as an abortive through the medium of the relation existing between the neck and body of the organ, by virtue of which impressions made upon the cervical nerves are reflected, through the spinal cord, upon the muscular fibres of the body and fundus, exciting them to contraction."

While, however, the latter—inflammation of the cervix—will most unquestionably produce abortion, it is more commonly the former—endouteritis—which, according to our author, exercises a morbid reaction on the ovum. When in a state of inflammation, the mucous membrane of the body may be altogether unapt to undergo transformation into the deciduous membrane, or only fitted for an imperfect and insufficient metamorphosis.

"In the first case," Dr. Miller observes, "abortion, and that speedily, must ensue; for the ovum cannot affect an attachment to the uterus, and may be degraded into a mole or a mass of hydatids; in the second, the disease may be communicated to the placenta, and through it to the fœtus, deranging its nutrition and variously affecting its growth, so as to produce, perhaps, even monstrosities."

That subacute inflammation of the lining membrane of the body and neck of the uterus, and other pathological conditions of that organ, though insufficient to prevent conception, may be among the occasional causes of abortion, no one who has examined the question with sufficient care will, we are persuaded, be inclined to doubt. We cannot, however, admit that abortion is so generally to be ascribed to a diseased state of the mucous membrane of the uterus, as Dr. Miller would seem to insist upon, inasmuch as we have not become convinced from the result of our observations, which have been by no means few in number, nor, we trust, superficially or carelessly conducted, that endo-uteritis, with its results, is a disease of so common occurrence as they are supposed to be by many respectable authorities, both in this country and in Europe. We are to recollect, also, as Dr. Miller himself pointedly inculcates, that disease of the womb is occasionally an effect rather than a cause of abortion.

After stating that abortion not unfrequently lays the foundation for future ill health, and especially for chronic disease of the uterus, affecting mostly the os and cervix, he remarks:—

"From my own experience, now somewhat extended, I am convinced that quite a large proportion of the cases of chronic inflammation and ulceration of this portion of the organ may be traced to abortion as their cause, as their history clearly reveals; for it often appears that, previous to an abortion, the health was unexceptionable, but since, though years may have elapsed, the patient has complained of uterine symptoms, which have been misunderstood until the true nature of the case was disclosed by a specular examination. That the os and cervix would be likely to suffer from abortion is no more than might, *à priori*, be expected, considering their organic condition at the time, and the amount of force that is required to overcome their resistance. Forced to dilate at a time when there is but little dilatability, lesion of structure is the not unfrequent consequence, which, though it may be slight, is sufficient to set up inflammation, which is all the more disposed to spread and take deep root on account of the travail so recently encountered. Whether this, my reasoning about the matter, be correct or not, still the fact remains, and is unimpeachable by clinical observation, that abortion is a frequent cause of cervical inflammation and its sequences, induration, hypertrophy, and ulceration."

The treatment of abortion is divided by Dr. Miller into the resistive, the palliative, and the prophylactic; or the means adapted, 1st, to avert the accident when threatened; 2d, to conduct it to as favorable an issue as possible when inevitable; and 3d, to guard against its occurrence by the removal of all causes liable to occasion it, or which may have already produced it in previous pregnancies of the same patient. Each of these means is very ably and fully discussed, and in accordance with the observations of the most reliable and recent authorities upon this branch of female therapeutics and hygiene. Under the head of prophylactic measures, Dr. Miller enters at great length into a consideration of the proper management of uterine disease in general—including cervical inflammation and ulceration, endo-uteritis, and uterine displacements.

Although we consider all this out of place in a systematic treatise on obstetrics, we cannot but award to the author the credit of having presented a highly instructive and discriminative account of the management of these affections, which will amply repay a careful perusal by the medical practitioner.

In regard to the recently much disputed question whether displacements of the womb are the invariable result, as one party maintains, of inflammation, or, as another affirms, while they are produced always by other causes than inflammation, are yet productive of the latter when they do occur, Dr. Miller considers both positions as too exclusive. He holds that though displacement of the uterus is not invariably caused by inflammation of the organ, it is so occasionally; while in other cases the inflammation detected by the metroscope is evidently the result and not the cause of the abnormal condition of the uterus.

Among the mechanical means suggested for the rectification of the several uterine displacements, Dr. Simpson's pessary comes of course under notice; in reference to it, Dr. Miller holds the following independent language, which will be endorsed, we are convinced, by every cautious and observant practitioner:—

"This kind of mechanical contrivance for holding the uterus in its proper situation appears to me to be the most unphysiological of all others that have been proposed. The uterus, in its natural state, possesses great mobility, amounting almost to locomotion, by which it is enabled to accommodate itself to the various disturbing influences by which it is surrounded. Pressed upon by the contractions of the diaphragm and abdominal muscles, during great muscular exertions, it descends towards the vulva; pushed up during coitus, its fundus is tilted forward and its cervix backward, while considerable distension of the urinary bladder necessarily retroverts it: in a word, it readily adapts itself to its circumstances, whatever they may be. Now, to rigidly fix such an organ upon a metallic axis, and leave it no possibility of escape under the thousand impulsions which it daily receives, is to place it in a more unnatural predicament than any malposition can possibly be. The intra-uterine pessary is, however, not more unphysiological than unpathological. Rarely is inflammation altogether absent in these displacements, and sometimes it is very intense; to irritate the uterus in such a state, by thrusting a foreign body into it to compel it to keep its place, is a species of surgery which would not be tolerated in the outward parts. That some of Professor Simpson's patients should not have been able to bear his pessary is not surprising; the wonder is that any of them could bear it, while it is a miracle that many of them got well.

"After all, the intra-uterine pessary fulfils its indication—albeit it performs its duty somewhat too sternly—that is, it keeps the uterus in the place allotted by obstetric authority, with its metallic axis coincident with the axis of the pelvic brim. And this is more than I am warranted to say in favor of any other pessary that has been recommended for retraversio uteri, not even excepting Prof. Meigs's elastic annular pessary, so much lauded by him.

"If it should be inferred from the foregoing remarks that I have but a poor opinion of pessaries, the inference would be logical and just; for though I have tried all kinds of pessaries, not excepting Dr. Simpson's, I have derived but little benefit from them, while they have been a fruitful source of vexation to myself and of annoyance to my patients. As, however, I have admitted the validity of the indication to restore the uterus to its natural position, it may be reasonably demanded, if pessaries are unavailing or mischievous, how is the indication to be accomplished? After I had discarded pessaries in the treatment of endo-uteritis complicated with retroversion, I was led to conjoin repeated replacement of the uterus by the sound, and cauterization of its cavity, while the organ is *in situ*, and by this method of treatment, which I may venture to call my own, have obtained more satisfactory results than by all other methods which I had previously tried."

The ensuing chapter (VI.) is devoted to a consideration of the flooding of advanced pregnancy and incipient parturition. Those sudden, profuse discharges of blood which occur toward the close of the period of utero-gestation, and always imply the separation, to a greater or less extent, of the placenta from the uterus, and the laceration of the utero-placental vessels, are among the most appalling accidents the obstetrician is liable to encounter, inasmuch as there is nothing which can befall a pregnant woman fraught with greater danger,—jeoparding the premature expulsion and death of the fœtus, as well also as the sudden death of the mother.

These floodings may be either *accidental* or *unavoidable*. The first resulting, in cases in which there is no abnormal position of the placenta, from the premature detachment, to a greater or less extent, of the latter, by mechanical violence, such as falls, blows, &c. Dr. Miller thinks it probable that an excited state of the circulation, especially in plethoric habits, may also, in some instances, suffice to bring about the same disastrous result. Irritation of the intestinal canal, whether induced by disease or the operation of drastic purgatives, that excite tenesmus and violent straining efforts, may also be reckoned among the causes of accidental uterine hemorrhage in the latter months of pregnancy.

Unavoidable uterine hemorrhage is that dependent on placenta prævia, or the implantation of the placenta in such a manner at the mouth of the womb, that the rupture of the utero-placental vessels is unavoidable as soon as labor sets in and the rapid development of the cervix and the dilatation of the os uteri commence. The latter form of flooding, which is the least manageable and most dangerous, is happily the less frequent of the two.

From the particular conditions attendant upon the occurrence of uterine hemorrhage during parturition, and particularly after delivery when the placenta is wholly separated and expelled, Dr. Miller concludes that in all cases of flooding, accidental or unavoidable, resulting from the partial or entire detachment of the placenta in the latter months of pregnancy, the chief, if not perhaps the only source of the hemorrhage, is the placental surface of the uterus itself.

In cases of placenta prævia it has been usually taught that the placenta is implanted either at or immediately over the os uteri, so that its attachments to the uterine parieties are necessarily destroyed by the dilatation, at the commencement of labor, of the uterine orifice. The inaccuracy of such opinion is clearly indicated by the author. He proves that, during the first six months of pregnancy, the cervix uteri preserves its cylindrical figure, and keeps both its orifices, but especially the superior, tightly closed. It is plain, therefore, that the placenta is implanted originally over the cervix, but attached, nevertheless, to the parieties of the inferior part of the body of the uterus. It is with the body, and the body only, that it forms an organic union by the reciprocal passage of blood-vessels between them. It is a matter of impossibility that in any case the placenta can be implanted on the internal surface of the uterine neck. The following is the correct explanation of the occurrence of hemorrhage in these cases, toward the close of pregnancy or immediately upon the occurrence of labor.

"During the first six months of pregnancy the uterus is developed at the particular expense of the fibres of the fundus of the organ, while during the last three months the fibres of the inferior part of the body are rapidly developed, insomuch that the further increase of size in the uterine cavity is acquired principally by the expansion of this portion of the uterus, as its pyriform shape in the early, and its perfectly avoidal shape in the latter months of pregnancy proves. This fact in respect to the development of the uterus, taken in connection with a fact already adverted to, viz. that the placenta has nearly completed its growth by the sixth month of pregnancy, affords a ready explanation of the occurrence of hemorrhage. When the placenta has its usual insertion, its development corresponds with that of the portion of the uterine walls upon which it is implanted, and there need be no hemorrhage; but when it is inserted over the neck, or even in its immediate vicinity, the matured placenta cannot follow the rapidly-expanding parieties of the uterus, and hence the stretching and rupture of the utero-placental vessels, and the unavoidable production of hemorrhage."

A very full and able account of the symptoms, course, and termination of flooding, is given in the second section of the present chapter, which need not, however, detain us.

The treatment of flooding Dr. Miller divides into the *medical* and the *obstetric:* including under the former all that can be done, either by hygienic measures or the administration of medicines, to control the discharge and prevent its return; and under the latter, the institution of labor and the delivery of the patient, when pregnancy can be no longer maintained without jeoparding her life.

When in cases of accidental hemorrhage the obstetric treatment is found to be unavoidable, Dr. Miller advocates the plan pursued by Puzos; in the annexed description of which we follow the translation given in the chapter before us.

"It is seldom that the loss of blood, produced by the detachment of the placenta, does not occasion more or less dilatation of the uterine orifice, the coagula about it acting as so many wedges that distend it and dispose it to yield. This incipient dilatation has a tendency to bring on labor, and is sometimes accompanied by slight pains. But inasmuch as the exhaustion and faintness arising from the loss of blood are opposed to the continuance of the uterine contractions, we must renew these when wanting, and increase them when too feeble. With this view, one or two fingers must be introduced within the os uteri, which is to be gradually opened by the employment of force proportioned to its resistance. These dilating efforts should be suspended from time to time, to allow intervals of rest. By this means the uterus is roused to action, labor-pains come on, and the membranes are rendered tense. The next object is to rupture the membranes without delay, to give escape to the liquor amnii, and bring about a diminution in the size of the uterus equal to the space the waters had occupied. The condensation of the uterus that succeeds the discharge of the liquor amnii presses the fœtus upon the os uteri, when stronger pains ensue, which, aided by the pressure of the fingers around the margin of the orifice, succeed in advancing the child. Meanwhile the blood that would otherwise have escaped is retained in the vessels by the contraction of the uterine fibres and the compression to which they are subjected. By this co-operation of nature and art, the delivery is greatly expedited, and we may enjoy the satisfaction of serving both mother and child, who must have been lost if left to nature alone, and who might have been destroyed by artificial delivery."

This same procedure of Puzos is recommended by Dr. Miller in cases of unavoidable hemorrhage from the partial presentation of the placenta. There can be no doubt that when skilfully and cautiously practiced, the manipulations referred to are adapted to the larger number of instances of accidental hemorrhage, and cases of partial placenta prævia, and will have the effect of bringing on regular and efficient contractions of the uterus, so as to hasten the delivery of the fœtus and suspend the discharge of blood. The procedure, to ensure its success and prevent injury resulting from it, demands, however, that the utmost judgment and care should be exercised in carrying it into effect; in the hands of a rash and inexperienced practitioner it is liable to be productive of far more injury than benefit.

In cases of placenta prævia in which the placenta is implanted centrally over the mouth of the womb, the artificial separation of the attachments of the after-birth to the inner surface of the uterus, with the view of arresting the hemorrhage which sets in immediately upon the commencement of labor, was proposed a few years since by Prof. Simpson; and the efficacy of the practice has been positively asserted by several distinguished obstetricians. Dr. Miller endeavors to show—and we believe that whoever will follow him in his examination of the subject will admit the correctness of his conclusion—that whether considered in a scientific point of view, or tested by its results in the cases in which it has been resorted to, so far as these have been made public, the artificial separation of the placenta, as proposed by Prof. Simpson in cases of placenta prævia, does not present that claim upon our confidence which would warrant a resort to it in the generality of instances.

Dr. Miller is still more opposed to the plan which, with a vertex presentation, has been pronounced by Rigby and others the only one that affords any chance for the preservation of the life of the mother, namely, turning the child, and its delivery by the feet. "If," he remarks, "delivery in this manner be undertaken too early, we may cruelly add to the sufferings of our patient and violently hurry on a fatal termination; if it be too long deferred, we dare not operate, lest we extinguish the flame of life, already flickering in its socket." Even when performed under the most favorable circumstances, turning is an operation attended always with extreme danger to the mother. On the whole, the feasibility and success of the operation of turning in placental presentation is not, according to Dr. Miller, such as to create any prepossession in its favor, but the reverse. The plan of treatment he recommends consists in "originating expulsive contraction of the uterus by the tampon or plug, and then puncturing the membranes, relying on the tampon to control the flooding, until the liquor amnii is evacuated."

This treatment, he remarks, is the one he has invariably employed, and, so far as the mother is concerned, with uniform success. It is true, he admits, his experience in cases of placenta prævia has not been large; it has been sufficient, however, to give him some acquaintanceship with their characters and management. In these cases, he remarks—

"The tampon is preferable to manual dilatation, as an oxytocic, because forced dilatation could not be practiced without necessarily still further detaching the placenta, giving rise to additional hemorrhage, all the more profuse on account of the non-purturient state of the uterus. Then, again, such manipulations would be objectionable because of the greatly more vascular and sensitive condition of the portion of the uterus contiguous to the os, which has been already mentioned as a reason why delivery by turning ought to be refrained from.

"In arousing the uterus to expulsive contraction the tampon acts, I suppose, through the channel that has been more than once indicated in the previous pages of this book, viz. irritation of the incident nerves of the cervix, leading to reflex action of the fibres of the fundus and body. Explain as we will, however, the fact is generally admitted that the tampon is competent to excite uterine contraction and bring on labor. Should it fail, (and what may not?) it may be reinforced by the puncture of the placenta as recommended by M. Gendrin, which, considered merely as a means of bringing on labor, is excellent and wholly unexceptionable; and it will be observed that I am not, just now, speaking of the restrainment of hemorrhage, but of the excitement of labor. No case can occur, I think, in which the tampon, aided if necessary by puncture of the placenta, will fail to bring on labor, in a longer or shorter time; and where the tampon alone is sufficient, and labor is regularly established by its instrumentality, either the placenta must be punctured to evacuate the liquor amnii, or the finger must be pushed up beyond its margin to rupture the membranes during a uterine pain. I have myself usually practiced the latter alternative, and always felt that my patient was safe when advanced thus far on the road to recovery.

"The supervention of labor—the evacuation of the liquor amnii—these, in their order, are the great bulwarks of a flooding woman, no matter where the placenta is implanted. It is a maxim in obstetrics that a *contracted* uterus cannot bleed; it might, I think, be amended and enlarged by adding that neither can a *contracting* uterus bleed when it is emptied of its waters—or, at any rate, if it bleed, the hemorrhage is no longer dangerous.

"Notwithstanding that the tampon may be generally depended on to restrain hemorrhage, it is certainly possible that it might disappoint our expectations, and then the evacuation of the amniotic fluid by placental puncture may be had recourse to, as an auxiliary, on the authority of M. Gendrin. Should this fail, and the hemorrhage threaten to destroy the mother ere uterine contraction can be excited, there can be no doubt of the propriety of separating and extracting the placenta, according to the letter of Dr. Simpson's proposal, viz. solely with a view to the safety of the mother, losing sight of the child, whose interests are altogether subordinate. Such a procedure excludes, of course, delivery by turning, by which, it has been shown, many maternal lives have been sacrificed that might have been saved, had the expulsion of the child been left to nature."

The causes of labor form the subject of the seventh chapter. The two sections of which are devoted to a consideration, the first, of the efficient, and the second, of the determinative cause of labor,—the chief efficient cause being the uterine contractions, aided as accessory causes, by the contractions of the diaphragm and abdominal muscles.

In respect to the determinative cause, or that which, at the termination of the normal period of utero-gestation, brings into operation the expulsive contractions of the uterus and its auxiliaries, Dr. Miller adopts the views advanced by Dr. John Powers, namely, that the uterine contractions are excited, when pregnancy has completed its term, by the irritation of the cervix, and especially of the os uteri, resulting from the contact, which then takes place, of the ovum with the latter. In evidence of the correctness of this explanation of the determinative or exciting cause of labor, Dr. Miller refers to the peculiar manner in which the uterine neck is unfolded during pregnancy,—it remaining unchanged until a very late period of gestation; until, in fact, a short time before labor sets in, so as to preclude the possibility of any contact between the fœtus and the os uteri occurring at an earlier period; excepting in cases in which the cervix is developed too soon or progresses too rapidly, when there will always be just ground for apprehending a premature expulsion of the fœtus. Another evidence of the correctness of Dr. Powers's theory is the fact that the rectum and bladder are both excited to expel their contents by irritation of their orifices.

The entire chapter is marked by great ability,—the views advanced in it, especially those in reference to the exciting cause of labor, are interesting and highly plausible; they cannot fail to demand a favorable consideration on the part of those interested in the physiology of obstetrics. That they are liable to many objections is very certain. The most important of these are very fully and candidly examined by Dr. Miller, who dismisses them as of insufficient force to invalidate the views advanced by Dr. Powers, supported as these are by facts and arguments of such acknowledged cogency.

The phenomena of labor is the subject next in order. (Chap. VIII.) These are clearly and accurately described in the order of their occurrence, while the comparative importance of each is indicated.

There has been some discussion as to the propriety of guarding from rupture the fœtal membranes until the os uteri has become fully developed and the head of the child has passed fully into the cavity of the pelvis,—it being maintained by some obstetricians, and those of the highest authority, that the pouch of waters, both before and after its partial protrusion at the uterine orifice, promotes the expansion of the os uteri, and thus promotes and facilitates the progress of labor; while others contend that after labor is fully set in the integrity or rupture of the fœtal membranes is a matter of perfect indifference, so far as the suffering and duration of the first stages of labor are concerned. Some have even gone so far as to assert that the early rupture of the membranes and discharge of the waters will often facilitate and shorten parturition. On this subject the following sensible remarks are made by Dr. Miller; they are in accordance with the views of the majority of the more authoritative obstetricians:—

"*First.* The integrity of the membranes before the pouch is formed is valuable, because the propelling force has then a more suitable medium wherewith to act on the cervix than any part of the fœtus would be. This medium is the waters inclosed by the membranes, which, adapting themselves to the shape and inequalities of the cervix, make more equable pressure on its fibres, and consequently subdue their resistance more equally; whereas any part of the child that can present is not so well adapted to distend the cervix equally, and hence, while some of its fibres may be numbed by pressure, others are not conquered, but provoked to inordinate resistance, thus retarding labor by the irregular contraction which is excited.

"*Secondly.* The integrity of the membranes, after the pouch is formed, is beneficial until the dilatation of the os uteri is considerably advanced, if not completed: because the pouch, though it does not cleave like a wedge, opens the portals for the egress of the child in the gentlest manner. Should it rupture before the orifice is prepared to allow the presenting part of the child to take its place, the ruder contact that ensues not unfrequently irritates the cervix to a renewal of its opposition, and labor is thus protracted and rendered more painful.

"*Thirdly.* The pouch serves, by its presence in the uterine orifice, the most sensitive portion of the neck, to sustain and enliven the propelling contractions, upon the principle of orificial irritation. By its agency, these contractions are, in proper time, rendered truly *expulsive*, and the auxiliary forces of the diaphragm and abdominal muscles called into action. When the pouch ruptures, the presenting part of the child takes its place, and keeps up the requisite grade of irritation, until the labor is completed. That this is no fancy sketch, the phenomena of shoulder presentations will abundantly prove. In these cases, the membranes frequently protrude in the form of a long, cylindrical purse, which inadequately stimulates the os uteri, and consequently the pains are feeble for an unusual length of time. When, at length, they rupture, if the shoulder is not ready to occupy the orifice, as often happens, there is *an entire suspension of the pains for several hours.*"

Although there is nothing absolutely new in the foregoing remarks, their general accuracy being recognized by nearly all skilful and observant practitioners, yet from their importance in a practical point of view, the necessity of urging them always upon the attention of young practitioners, and the very excellent manner in which they are put by the author, we have been induced to copy them.

It is in this connection that Dr. Miller discusses the mechanism of labor, an intimate acquaintance with which is an essential prerequisite to the formation of the practical obstetrician. With entire ignorance, or imperfect and confused conceptions of the manner in which the fœtus, in the several presentations and positions it may assume, is transmitted through the pelvis,—its course, the successive changes which occur in the position and direction of the presenting part, as it is gradually propelled from the womb into the world,—the practitioner may, perhaps, stand by with folded arms during the progress of an easy, natural labor, and witness its favorable termination, the result probably of his very non-interference; but, in cases in which anything intervenes to render it tedious, difficult, or dangerous, or in which, from any cause, there is a necessity for prompt manual or instrumental interference, he can neither act with decision or skill, but, groping in the dark, is more liable, by his interference, to injure than to benefit mother and child.

The description given of the mechanism of labor by Dr. Miller is, upon the whole, clear and correct. His explanatory and critical remarks, under the head of each presentation, are replete with good sense, and may be studied with profit.

The classification of the several fœtal presentations and positions adopted by the author, is that of M. Dugès. He has introduced a slight variation of their nomenclature, to which no particular objections can be offered. The great desiderata in the terminology of this and every other branch of professional science are precision, simplicity, and shortness. These may, however, be sacrificed, to a certain extent, for the sake of uniformity. Nothing is more annoying to student and practitioner than to find each of the authors who treat upon the same subject adopting a classification and terminology of his own.

The ninth chapter is devoted to the diagnosis and prognosis of labor. The first, as it has for its object the discrimination between labor and all other conditions of the sexual organs and general system of the female, with the differences among labors, growing out of the accompanying circumstances, especially the fœtal presentations and positions, is invested with no trifling degree of importance, and fully warrants the extension that Dr. Miller has given to its consideration.

There is no doubt that in reference to the prognosis of labor,—its probable duration in any given case, its favorable or unfavorable termination as it respects the mother or child, or both, if left to the unaided powers of nature, and the necessity and period for manual or instrumental interference, certain general rules may be laid down, in the highest degree useful for the guidance of the practitioner. After all, however, these rules are rather suggestive than absolute in their application to each case as it occurs. It is from a close study of the principles of obstetrics, the mechanism of labor, the forces by which it is accomplished, the nature and comparative frequency of the various causes by which its progress may be retarded, and its completion, without the interference of art, prevented, in connection with that peculiar tact which can be acquired only by careful clinical observation, that a facility in arriving at a skilful and reliable prognosis of labor in any case can be acquired. The sections devoted to the subject in the chapter under consideration are clear, explicit, and instructive; they are well adapted for the use of the obstetrical student.

The remaining chapters of the treatise, from the tenth to the sixteenth inclusive, are devoted to the management, or, as Dr. Miller expresses it, the treatment of labor. We shall not stop to notice the inconvenience attendant upon the very clumsy arrangement which Dr. Miller has adopted in this portion of the work; but, as we run over the seven concluding chapters, notice such subjects comprised in them as strike us in consequence of their intrinsic importance or the manner in which they are treated by the author.

Dr. Miller enumerates as the principal retarding and impeding causes of labor: in the first stage, obliquity of the uterus; the inefficient or impeded action of the organ; rigidity of the os uteri: and in the second stage, inefficient and impotent action of the womb.

The proper indication in cases of obliquity of the uterus is the prompt restoration of the os uteri to its natural position. This may generally be accomplished by rest in the horizontal position; the female lying on the side opposite to that toward which the fundus uteri is inclined. When anterior obliquity is present to an extent sufficient to interfere with the expulsive action of the uterus, a bandage properly applied around the abdomen will greatly assist in the restoration of the womb to its proper position.

When strict attention to posture, continued for a reasonable time, fails to correct the malposition of the uterus, and there is in consequence evident retardation of the progress of labor, Dr. Miller advises—

"To hook the os uteri by inserting the extremity of the finger within its orifice, and draw it toward the centre of the pelvis, in the intervals of the pains. When a pain comes on, its tendency to relapse to its former position is to be resisted with as much force as can be safely employed. If this tendency is too powerful to be resisted, the finger must yield to it; but as soon as the pain ceases, bring back the os uteri to the centre, and again endeavor to maintain it there during the next pain. By cautiously and gently, but perseveringly acting thus, the os uteri will, after a succession of centripetal and centrifugal movements, be restored to its proper place, and, the parturient forces having been brought to bear upon it, its dilatation will be effected as speedily as in ordinary cases."

Awkward and unskilful attempts at rectification of the obliquity may, Dr. Miller admits, produce inflammation of the uterus; but this, he adds, is chargeable to the operator, not to the operation, which need not cause pain, much less any such serious consequences.

"For my own part," he observes, "I can safely declare that no mischievous effects of any kind have ever resulted, in my practice, from such tractions on the os uteri as have been recommended; and the testimony of Dr. Dewees is equally decisive in regard to their safety and efficacy. Nay, this eminent practitioner deemed them of so much importance as to advise the introduction of the entire hand, well lubricated, into the vagina, in order to get hold of the os uteri with the finger, when it cannot be reached by a well-directed search in the ordinary way; and, under the circumstances that he has specified, I should not hesitate to follow his advice, although I have not as yet had occasion to do it."

When labor is rendered tedious by the inefficient action of the uterus, attention should be directed to remove, if possible, any morbid condition of the system that may be present. If the pulse be full and strong, venesection will be proper; if the bowels are confined, they should be evacuated by an enema, or some mild, active purgative, as castor oil. If these means fail, or should they not be indicated, Dr. Miller directs the irritation of the uterine orifice by means of the finger, for the purpose of bringing on more efficient contractions of the uterus. He has had, he informs us, abundant experience in the employment of such manipulation, in the cases indicated, and, while he has seen no evil result from it, when properly practiced, he has often been surprised and gratified at its prompt and beneficial effects. "Not unfrequently a few movements of the fingers are sufficient to impart such energy and aim to the uterine contractions that the waters begin to gather, as the phrase is, and cause the membranes to protrude." Labor proceeding regularly and actively to a favorable termination.

During the first stage of labor, one of the most common causes of impeded uterine action is the premature rupture of the membranes. Dry labors, as the nurses term them, are proverbially tedious and painful. When, in such cases, from the rude contact of the presenting part of the fœtus with the os uteri, before this has become sufficiently relaxed to dilate promptly, it becomes hot, dry, tender to the touch, rigid and swollen, the free detraction of blood, when there is nothing in the constitution and general condition of the patient to forbid it, should never be neglected; following the bleeding immediately by a full, or rather large dose of opium, will, in general, be found to be productive of the best effects. Dr. Miller believes that in these cases the judicious employment of the fingers in aid of the uterine contractions is often the best means of facilitating labor. Not for the purpose of exciting uterine contractions, for these are already too strong, nor to stretch open the os uteri, but simply to press upon the margin of the latter during the pains, "in order that their counter-pressure may keep it in as firm contact with the head as the rest of the cervix, and the orifice be thus brought within the pale of the dilating influence of the uterine contractions. Both Hamilton and Gooch highly recommend this practice; but their object is to *push up* the edge of the orifice over the head of the child, to liberate the band of the cervix, supposed to be incarcerated,—a condition which, if it really existed, could scarcely be reached by such a procedure."

In cases of inordinate rigidity of the os uteri, Dr. Miller recommends the ordinary treatment,—copious blood-letting, opiate enemata, tartarized antimony administered with a view to its nauseating and relaxing effects, with tepid baths or demi baths. He advises, also, the local application to the os uteri of the extract of belladonna or of stramonium. If these means fail in causing a sufficient relaxation of the os uteri, and the cervix, instead of becoming obliterated, is forced down before the advancing head of the child, it becomes necessary to raise the os uteri in the intervals of the pains, and to support it during the continuance of the pain. To this end—

"The index finger is to be applied just underneath the anterior lip of the os uteri, and, with its edge or palmar surface, pressure is to be made in the intervals of the pains, so as to push up the os uteri as high as possible, or the extremities of *two or three of the fingers may be used* in the same way. When a pain comes on, the tendency to descent is to be resisted, unless this be so strong as to require more force than it would be prudent to employ; in that case, the finger must gradually relax its counter-pressure and allow the descent to take place. But as soon as the pain goes off, the os uteri is to be pushed up again, and its descent is again to be resisted during the next pain. In this manner, acting with gentleness and caution, but at the same time with firmness and perseverance, the os uteri must be supported until it is sufficiently dilated to allow the head to execute its rotatory movement and emerge from under the pubis.

"The principle upon which this manœuvre acts does not appear to be well understood, even by those who have practiced it. The support given to the os uteri prevents it from prolapsing, to be *backed*, if the expression will be allowed, by the floor of the pelvis, and places it in a position that will permit a portion of the head to become insinuated within it during a pain. The finger is not used to stretch the os uteri, as many writers direct, but to hold it up that it may be dilated by the head, which can then be pushed by the uterine contractions lower than the level at which the os uteri is held. The head dilates the os uteri far better than the finger could, because it acts upon the whole extent of the cervix, whereas the finger could act only on the circle of the os uteri."

When, in the second stage of labor, the dilatation of the os uteri is completed, and the pains become expulsive, or even in the absence of efficient expulsive pains, in order to bring them on, it is the practice of Dr. Miller, and we believe the practice to be a correct one, to rupture the membranes and allow the waters to escape, in all cases without exception.

It is in the second stage of labor, according to our author, that the production of anæsthesia exerts its most beneficial effects by abolishing the pains of child-birth, which are then the most severe, and by quelling the violent nervous agitation which is now at its height, and at the same time removing that "certain painful looking-for" of anguish still to be endured, which is often more torturing to the parturient female than the actual pangs of parturition.

Dr. Miller informs us that he has been in the habit of administering chloroform for nearly ten years, in natural as well as in difficult labors; during which period he has given it in many hundred cases, but he is not aware that, in a single instance, any injurious effect has followed its employment.

"I have occasionally known it to diminish the energy of the uterine contractions, and lengthen the intervals between them to such a degree as to make it expedient to suspend its use for a time or altogether discontinue it. 'But in these exceptional cases no harm resulted, and when the anæsthesia passed off—which it did in a short time—the labor resumed its course and progressed as though it had suffered no interruption."

Dr. Miller believes that the beneficial influence of anæsthesia is not confined to the parturient state, but extends also to the puerperal. His patients who had previously been delivered without chloroform have often remarked, without prompting or questioning, that after they had been subject to its influence, "they felt very much better than in their former confinements, having little or none of the muscular soreness and stiffness, or the fatigue and exhaustion which they were before accustomed to feel;" while Dr. Miller is convinced also that their convalescence is altogether better.

Inasmuch as we possess in the *ergot* a very certain agent for the production of uterine contractions, the question very naturally presents itself, May it not be beneficially resorted to, in order to overcome inefficient action of the womb, when this occurs during the second stage of labor? Whenever the os uteri is fully dilated, the vagina and vulva are relaxed and moist, the presentation being natural, or such as offers but slight impediment to the birth of the child, and there is at the same time no disproportion between the presenting part and the pelvis, there can be no doubt but that under such circumstances the ergot may be safely employed to excite uterine contractions, when these are suspended, irregular, or inefficient; but in no instance in which any of the foregoing conditions for a prompt termination of the labor, under efficient uterine contractions, are absent, should the employment of the ergot be permitted. In the absence of either of them, the powerful and continued contractions of the uterus produced by the ergot will most certainly prove destructive to the child, while at the same time they will endanger also the life of the mother. When, in any case, there is the slightest doubt as to the entire safety of the employment of ergot, it should invariably be abstained from.

Under such circumstances, Dr. Miller recommends, with an endorsement of their efficiency, a resort to the same manipulations that are recommended by him in the case of insufficient action of the uterus occurring in the first stage of labor.

We will here repeat the remark we have already made, which is, that in the hands of the cautious and experienced practitioner, the manipulations just referred to, as well as others recommended in different parts of the treatise under consideration, will very generally be attended with unquestionably good results; but when heedlessly resorted to by the rash and inexpert, they may, on the contrary, be productive of serious mischief.

When, in the course of labor, impotent action or complete inertia of the uterus sets in, as the result of exhaustion from the long continuance of the parturient efforts, the question as to the necessity of artificial delivery comes up for consideration and decision. Its correct solution must be founded upon the unquestionable evidences present of uterine impotency, the condition of the patient, and the probabilities of the labor being terminated with perfect safety to both mother and child without manual or instrumental aid. It would be unwise to wait until the last moment before resorting to the latter,—until the urgency is extreme and the chances of success materially diminished. Dr. Miller lays down the correct general rule, that in all cases in which the evidences of the impotency of the uterus to accomplish the delivery of the child are unquestionable, the earlier that artificial delivery is resorted to, provided it can be effected with facility and safety, the better it will be for both mother and child.

"Suppose, for example," he remarks, "the head of the child is presenting and has ceased to advance, while the uterus has evidently become impotent; suppose, moreover, this head is within easy reach of the forceps, and can be delivered without risk or additional pain to the mother, what would be the use of waiting until we are driven to the operation? But, if delivery be not so easily and safely practicable, prudence requires that it should be deferred until the necessity of it is more pressing,—so pressing that, in our judgment, it is better to incur whatever risk the operation may involve than wait longer."

On the subject of instrumental delivery,—the particular cases and conditions of labor in which a resort to it is proper and prudent, with the manner in which it is to be performed, according to the particular presentation and position of the child, and, also, the particular circumstances which, in each, preclude the possibility of the labor being completed with safety to the mother and child by the unaided action of the uterus,—the teachings of Dr. Miller are, upon the whole, particularly sound and judicious. His instructions for the application and management of instruments under the several circumstances in which they may be demanded are plain, simple, and correct, and as clear as it is possible to render such instructions by printed descriptions, aided by well-executed pictorial illustrations.

In cases of puerperal convulsions, Dr. Miller considers that there are two leading indications to be fulfilled, namely, to guard, by the judicious detraction of blood, the important vital organs, especially the brain, against the danger of overwhelming congestion during the convulsive paroxysms; and, secondly, to relieve promptly the womb by the forced delivery of the child. He very properly condemns the profuse and repeated bleedings, so commonly recommended in cases of puerperal convulsions, as both useless and mischievous: he would restrain the emissing of blood within such limits as are sufficient to place the blood-vessels in a condition to bear the stress laid upon them by the successive convulsive paroxysms. The fulfilment of the second indication, the early relief of the uterus from its contents, he believes to be of such imperative necessity that he lays it down as the duty of the obstetrician, in cases in which the condition of the os uteri will not permit the forceps to be applied, to deliver by craniotomy, whether we are certain or not of the death of the child, and thus get rid without delay of "the cause that sustains the convulsions," without the necessity of allowing hours to pass by while waiting for the os uteri to become sufficiently dilated to permit of delivery by the forceps or by turning.

"It is not recommended," he observes, "to have recourse to craniotomy in every case of parturient convulsions so soon as the operation is practicable. Not so; for it may be that there is no such urgent and instant necessity as to justify the operation. But if it be otherwise, and there are just grounds for our worst apprehensions, while from the tedious manner in which the dilatation of the os uteri is going on it is wholly uncertain how long we must wait before delivery can be accomplished by the forceps, then the perforator should be used, even if the child be alive."

We most positively dissent from both the positions here assumed by Dr. Miller, namely, that the presence of the child in utero is always the cause by which the convulsions are kept up, and that there is, in consequence, an imperative necessity for immediate delivery, even when to effect it the life of the child must be sacrificed.

We have very seldom known the convulsions to cease upon the expulsion of the child, while some of the most violent cases we have ever met with did not commence till after delivery. When convulsions occur previous to or during labor, with a favorable presentation, we are always able, by the judicious use of anæsthetics, so far to suspend the convulsive paroxysms as to allow sufficient time for the natural progress and termination of parturition; or, at least, for the os uteri to become relaxed and dilated to an extent sufficient to allow of delivery being accomplished by the forceps, and that without increasing in the slightest degree the danger of the mother.

The operation of turning and delivery by the feet, let it be ever so cautiously and skilfully performed, is one that is admitted on all hands to be liable to entail upon the parturient female no trifling danger, either immediate or remote. In view of this fact, Dr. Miller would restrict it absolutely to cases of shoulder presentation. Here, as the only operation adapted to relieve the case of its difficulties, he would have it invariably resorted to so soon as the character of the presentation is detected and the os uteri is in a condition to permit the safe and easy introduction of the hand. Dr. Miller is persuaded that cephalic version, even when practiced according to the correct and scientific plan pointed out by Dr. Wright, of Ohio, is of very limited application, it being only practicable in those cases in which the labor is in its first stage, the os uteri barely dilated, the membranes entire or but recently ruptured, the uterine contractions not very powerful or decidedly expulsive, and the shoulder not yet impacted in the pelvis. The following are his conclusions in regard to the comparative merits of the two plans of version,—the cephalic and podal:—

1. "In simple cases of shoulder presentation, either cephalic or podical version may be practiced, the former being preferable (provided it can be accomplished) on account of the greater probability of saving the child.

2. "While cephalic version is not absolutely impracticable in complex, and even complicated cases of shoulder presentation, it is more difficult of performance and fully as dangerous, both to mother and child, as podalic version in one or other of its forms.

"But granting, for the argument's sake, that cephalic version is in its ultimate results as eligible as podalic version, still I should contend that in complex, much more in complicated cases, it ought never to be had recourse to, because the sufferings of the patient are uselessly protracted by it. Even if there be not exhaustion of the uterus, it is impossible to know beforehand, in any such case, whether the child will be expelled by the natural powers or require to be delivered by instruments, thus subjecting the patient to the combined hazard of manual and instrumental delivery."

The closing topic of the last chapter of the treatise is the cause, consequence, and proper management of flooding subsequent to delivery. On this subject, the views and practical directions of the author are particularly sound and judicious. It is true that his estimate of the value of ergot as a hæmostatic in cases of post partum hemorrhage is very much below what our experience of its effects has taught us to form of it. Dr. Miller considers its operation to be too precarious to justify us in relying on it to the neglect of more certain and reliable means.

Flooding, when it occurs subsequently to delivery, is always of too formidable a character to warrant the neglect of any of the means adapted to arrest the discharge of blood, or which may concur with other remedies in effecting this object,—of whatever agent within our reach that will produce prompt, complete, and permanent contraction of the uterus. This is, in fact, the chief indication to be fulfilled; until it is, the patient is at any moment in danger of a return of the flooding to even a greater extent than previously. We are acquainted with no other agent that will so certainly excite uterine contractions with the same degree of promptness and to the same extent as ergot. So important a remedy do we esteem it to be for the control of post partum flooding, that we must insist upon the propriety of its administration in every case in which, subsequent to delivery, uterine hemorrhage is either threatened or has already commenced.

In the treatment of post partum flooding, Dr. Miller very properly condemns the use of the tampon, as a most hazardous expedient. It may control the issue of the blood externally, while this continues to pour from the vessels and collect in the uterine cavity, endangering the life of the patient to as great an extent, perhaps, as though permitted to flow externally. Let the blood, says Dr. Miller, have an unobstructed channel, the danger of our patient will be then more apparent, and we incited to more earnest efforts to save her from impending death.

We here close our notice of the work before us. Of its general value we have formed, it will be perceived, a very high estimate. We bespeak for it such a reception on the part of the American medical profession as will render, before long, the preparation of a second edition necessary; in which the omissions we notice in the present can be supplied, and the treatise rendered complete in all its parts; while, at the same time, the few flaws which now mar its general excellence can readily be removed.

